# Survivin antagonizes chemotherapy-induced cell death of colorectal cancer cells

**DOI:** 10.18632/oncotarget.25600

**Published:** 2018-06-12

**Authors:** Anke Rauch, Annemarie Carlstedt, Claudia Emmerich, Al-Hassan M. Mustafa, Anja Göder, Shirley K. Knauer, Michael Linnebacher, Thorsten Heinzel, Oliver H. Krämer

**Affiliations:** ^1^ Center for Molecular Biomedicine, Institute of Biochemistry and Biophysics, Department of Biochemistry, Friedrich Schiller University Jena, 07745 Jena, Germany; ^2^ Leibniz Institute on Aging, Fritz Lipmann Institute, 07745 Jena, Germany; ^3^ Department of Toxicology, University Medical Center of the Johannes Gutenberg University Mainz, 55131 Mainz, Germany; ^4^ Department of Molecular Biology, Centre for Medical Biotechnology (ZMB), University of Duisburg-Essen, 45141 Essen, Germany; ^5^ Department of General Surgery, Molecular Oncology and Immunotherapy, University of Rostock, 18057 Rostock, Germany

**Keywords:** ATR, irinotecan, oxaliplatin, p53, survivin

## Abstract

Irinotecan (CPT-11) and oxaliplatin (L-OHP) are among the most frequently used drugs against colorectal tumors. Therefore, it is important to define the molecular mechanisms that these agents modulate in colon cancer cells. Here we demonstrate that CPT-11 stalls such cells in the G_2_/M phase of the cell cycle, induces an accumulation of the tumor suppressor p53, the replicative stress/DNA damage marker γH2AX, phosphorylation of the checkpoint kinases ATM and ATR, and an ATR-dependent accumulation of the pro-survival molecule survivin. L-OHP reduces the number of cells in S-phase, stalls cell cycle progression, transiently triggers an accumulation of low levels of γH2AX and phosphorylated checkpoint kinases, and L-OHP suppresses survivin expression at the mRNA and protein levels. Compared to CPT-11, L-OHP is a stronger inducer of caspases and p53-dependent apoptosis. Overexpression and RNAi against survivin reveal that this factor critically antagonizes caspase-dependent apoptosis in cells treated with CPT-11 and L-OHP. We additionally show that L-OHP suppresses survivin through p53 and its downstream target p21, which stalls cell cycle progression as a cyclin-dependent kinase inhibitor (CDKi). These data shed new light on the regulation of survivin by two clinically significant drugs and its biological and predictive relevance in drug-exposed cancer cells.

## INTRODUCTION

Colorectal cancer is the third most frequently occurring tumor in men and women. About one million cases are diagnosed per year and this cancer is the fourth most common cause of tumor-related deaths [1]. Oxaliplatin (L-OHP) and irinotecan (CPT-11) in combination with 5-fluorouracil are standard treatment options for primary and metastasized colorectal cancer [2].

L-OHP, a diaminocyclohexane-platinum complex, forms adducts with d(GpG) in DNA in a cell cycle-independent manner [3, 4]. The resulting inter- and intrastrand crosslinks block DNA replication and transcription, with interstrand crosslinks (ICLs) being the most cytotoxic DNA aberration [3, 4]. The nucleotide excision repair (NER) system and the homologous recombination pathway (HR) or translesion polymerases remove and repair such DNA lesions [3, 5, 6]. NER comprises two arms, global genomic repair (GG-NER) and transcription-coupled repair (TC-NER). While the recognition of platinum-DNA adducts by GG-NER triggers p53- and caspase-3-dependent apoptosis, TC-NER deficiency increases sensitivity to platinum compounds [3, 5].

CPT-11 inhibits topoisomerase 1, which cleaves single strand DNA to ease tension that arises during the replication and the transcription of DNA. Consequently, single and double strand DNA breaks occur from torsional stress, inhibited DNA re-ligation, and an ensuing replication fork collapse [7, 8]. The HR pathway repairs CPT-11-induced DNA lesions [7, 8].

The sensor checkpoint kinases ataxia telangiectasia mutated (ATM) and ATM and RAD3-related (ATR) are among the first factors that are phosphorylated in cells with double and single strand DNA breaks [9, 10]. Phosphorylation of ATM occurs at S1981 [11] and several other serine and threonine residues [12]. ATR undergoes phosphorylation at T1989 and S428 [9] and can also act as an upstream activator of ATM [13]. Subsequently, ATM/ATR phosphorylate their downstream substrates checkpoint kinase 1 and 2 (CHK1/CHK2) at several residues, including S317 (CHK1) and T68 (CHK2) [14, 15]. These enzymes and their targets integrate cell cycle progression, DNA repair, and cell death upon irreversible DNA damage. The tumor-suppressive transcription factor p53 is a substrate of checkpoint kinases and a most critical mediator of these processes [10]. Checkpoint kinases control cell death induction by L-OHP and CPT-11 to a variable extent. ATR-CHK1 and ATM-CHK2 signaling cascades protect colon cancer cells from CPT-11 [7, 16–18]. In contrast, L-OHP-resistant colon cancer cells have low levels of ATM [19]. The roles of ATR and CHK1 in colon cancer cells exposed to L-OHP are unclear, but data collected with other cell types suggest a minor role of ATR-CHK1 signaling for L-OHP-induced DNA damage and cell death [20].

Pro-apoptotic effects of chemotherapy, γ-irradiation, and targeted therapy are frequently blunted by defects in the apoptosis machinery of cancer cells [21, 22]. The underlying mutations could arise during the transformation step, when normal cells have to cope with oncogenic stress, and/or as a clonal amplification of more robust cells during therapy. The transcription factors p53 and NF-кB, as well as several of their target genes, are among the factors that are prone to mutations and dysregulation during tumorigenesis [21–23]. Their target genes include B cell lymphoma-2 (BCL2) family members and the inhibitors of apoptosis (IAPs) protein, for example survivin, which is encoded by the *baculovirus IAP repeat containing-5 (BIRC5)* gene [24–26]. An inactivation of such proteins in cancer stem cells could be a possibility to eliminate colon tumors effectively [21, 22]

A better identification and understanding of the molecular mechanisms that chemotherapeutics induce and how tumor cells develop drug resistance will improve cancer therapy. Our work shows that L-OHP and CPT-11 affect cell cycle arrest, checkpoint kinase signaling, and apoptosis differentially. Whereas L-OHP suppresses the expression of the anti-apoptotic protein survivin, CPT-11 fosters its induction. We further demonstrate that a p53/p21-dependent suppression of survivin is essential for cytotoxic effects of L-OHP. In contrast, CPT-11 stabilizes survivin in a ATR-dependent and p53-independent manner and an inhibition of survivin can accentuate pro-apoptotic effects of CPT-11.

## RESULTS

### L-OHP and CPT-11 alter cell cycle progression

**Figure 1:**
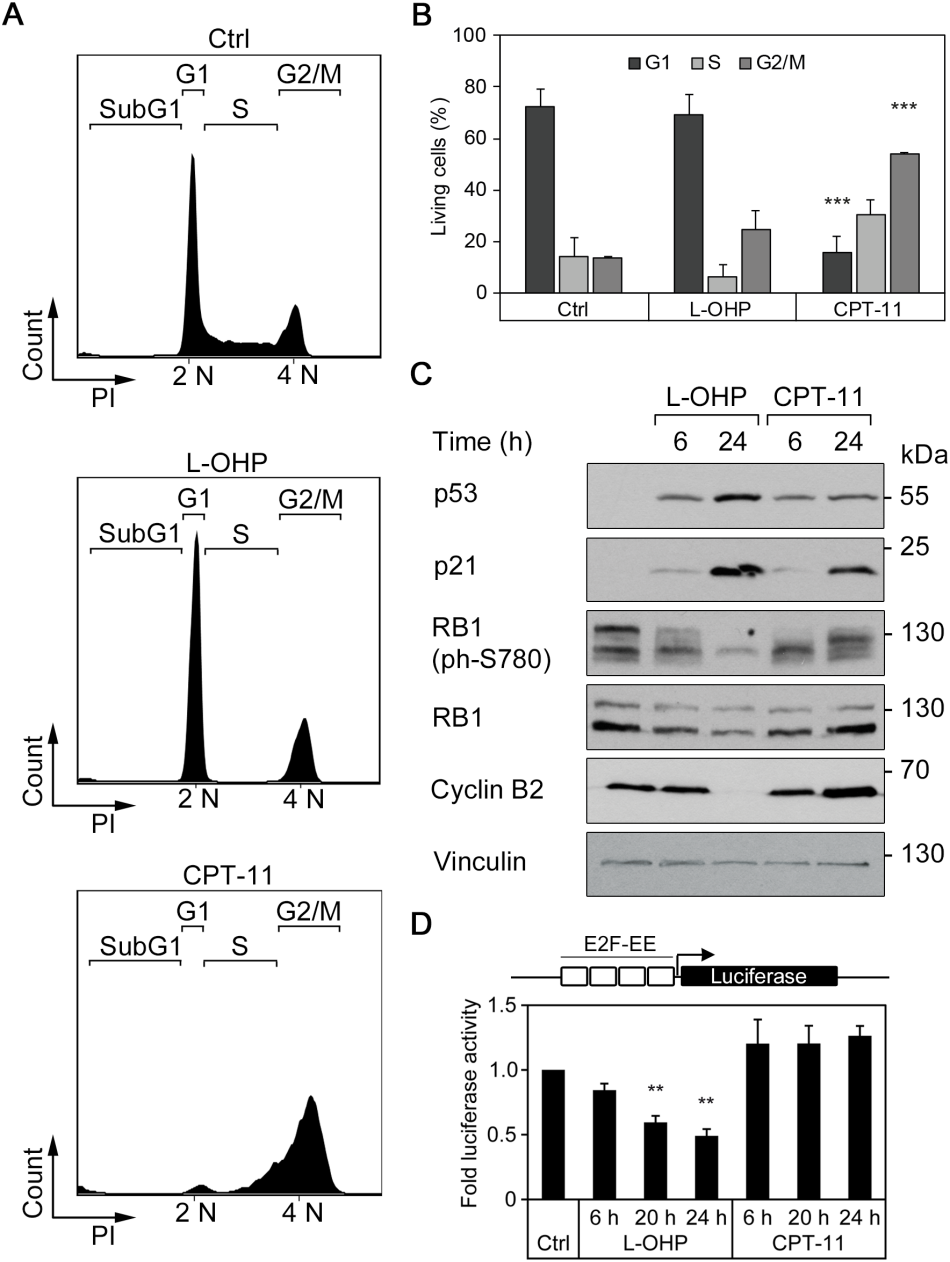
L-OHP and CPT-11 affect cell cycle behavior in human colorectal cancer cells HCT116 **(A)** Representative cell cycle profiles after treatment with 5 μM L-OHP, 10 μM CPT-11 or DMSO (Ctrl) for 24 hours. Shown are subG_1_, G_1_, S and G_2_/M-populations according to their cellular DNA content (n = 3). **(B)** Relative numbers of living cells in the G_1_-, S- or G_2_/M-phase of cell cycle after treatment for 24 hours. Data represent mean ± SD of three independent experiments (^**^p < 0.01, ^***^p < 0.001). **(C)** Western blot analysis using antibodies against p53, p21, RB1, phosphorylated RB1 as well as cyclin B2 (n = 3); vinculin serves as loading control. **(D)** E2F-dependent activation of luciferase reporter construct after treatment with L-OHP or CPT-11 for 6, 20, and 24 hours (^**^p < 0.01, n = 3).

In order to analyze how L-OHP and CPT-11 dysregulate cell cycle progression and the expression of pro- and anti-apoptotic factors, we treated HCT116 colorectal cancer cells for 24-48 hours with these drugs. We analyzed the cells by flow cytometry to determine their cell cycle profiles. Cells were fixed, permeabilized, and stained with the DNA dye propidium iodide (PI). We excluded dead cells that contain less than 2N DNA content due to DNA fragmentation in the cell cycle analyses. The untreated cell populations typically consisted of about 72% of cells in the G_1_-phase, whereas the S- and G_2_/M-phases each contained about 14% of the populations. After 24 hours, L-OHP reduced the number of S-phase cells to 6.0% (Figure 1A and 1B), indicating stalled cell cycle progression from G_1_- to S-phase. In contrast, CPT-11 caused a significant reduction of the G_1_-population and most cells accumulated in the S- and G_2_/M-phases (Figure 1A and 1B).

Next, we investigated the expression of cell cycle regulatory proteins in L-OHP- and CPT-11-treated HCT116 cells. We analyzed the levels of p53 and its target gene p21 (p21^WAF/CIP1^; a cyclin-dependent kinase inhibitor), total and phosphorylated retinoblastoma-1 (RB1) protein levels, and cyclin B2. Western blot analyses showed that p53 accumulated after 6 and 24 hours in HCT116 cells treated with L-OHP and CPT-11 (Figure 1C). Accordingly, both drugs induced p21, with L-OHP being a stronger inducer than CPT-11. Untreated asynchronously cycling cells showed RB1 with various extents of phosphorylation (Figure 1C). L-OHP reduced RB1 phosphorylation at its serine residue 780 (S780). A 24-hour treatment with CPT-11 induced less p21 and after 6 and 24 hours, CPT-11 caused hyperphosphorylation of RB1 at S780 (Figure 1C). Cyclin B2 accumulates in G_2_/M-phase [27, 28]. Consistent with their divergent abilities to stall cells mainly in the G_1_- or the G_2_/M phases of the cell cycle, CPT-11 induced and L-OHP repressed the levels of cyclin B2 (Figure 1C).

Since E2F transcription factors are critical regulators of cell cycle progression, we analyzed their activity by measuring the activity of an E2F-dependent luciferase reporter. After 6 to 24 hours, L-OHP suppressed E2F-dependent reporter gene expression increasingly and CPT-11 induced the E2F-dependent reporter slightly (Figure 1D).

We conclude that L-OHP and CPT-11 exert variable effects on the cell cycle and its molecular regulators in colorectal cancer cells.

### L-OHP and CPT-11 induce different levels of replicative stress and DNA damage

**Figure 2:**
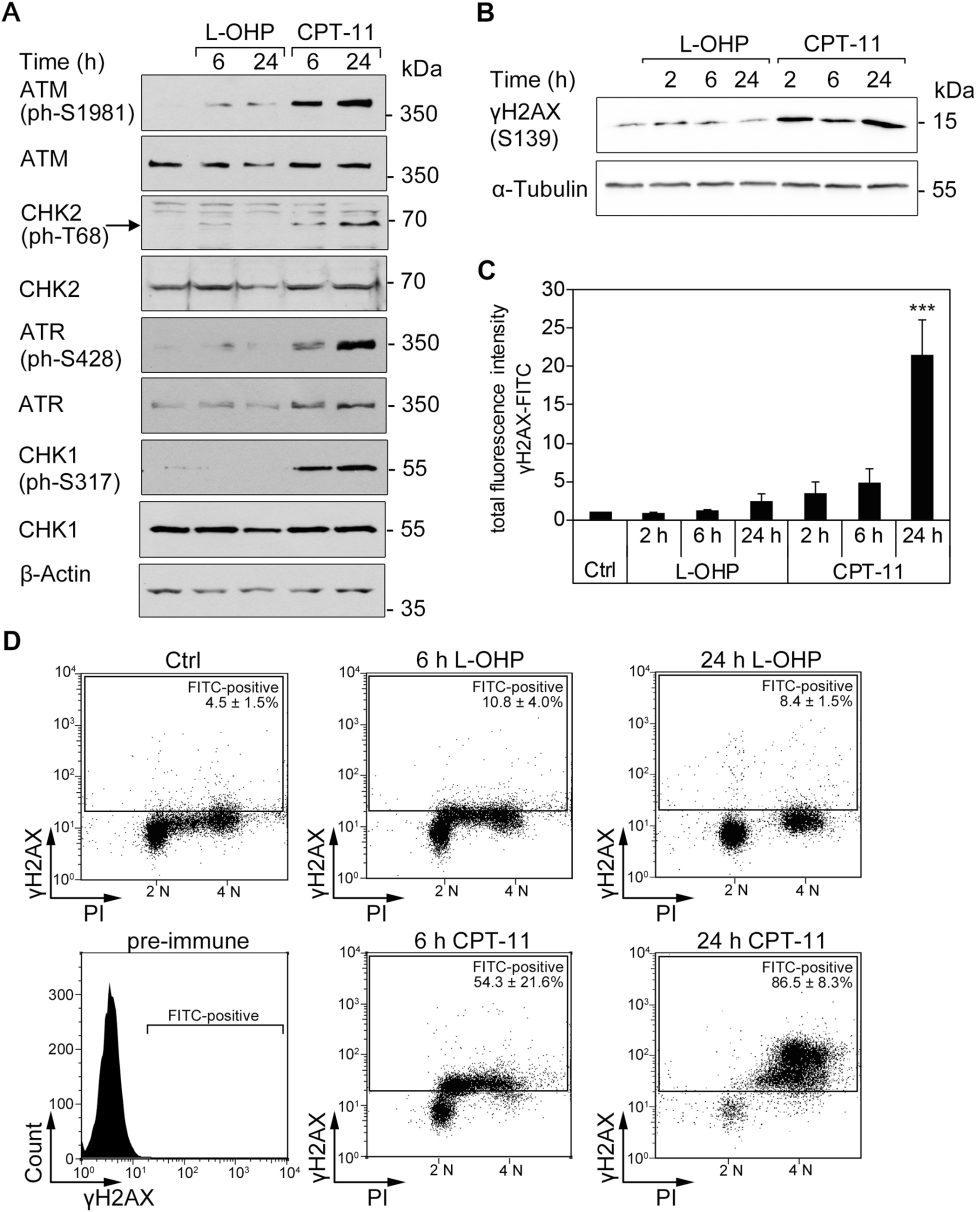
DNA strand breaks are induced by CPT-11, but not after L-OHP in HCT116 cells **(A)** Western blot analysis of whole protein levels and phosphorylation patterns of ATM, CHK2, ATR, and CHK2 (n= 3); β-actin serves as loading control. **(B)** Western blot analysis and immunostaining of cellular γH2AX (S139); α-tubulin serves as loading control. **(C)** Intracellular immunostaining of γH2AX protein levels with FITC-conjugated antibody and flow cytometric analysis of the cellular fluorescence intensity. Depicted is the total fluorescence intensity of FITC-positive cells after 2, 6, and 24 hours treatments with 5 μM L-OHP, 10 μM CPT-11, or solvent control (^***^p < 0.001, n = 4). **(D)** Comparison of γH2AX-FITC levels and DNA content of DAPI-stained cells. Depicted is the mean number of FITC-positive cells (n = 4).

To further characterize how L-OHP and CPT-11 affect colorectal cancer cells, we probed for markers of DNA damage and associated signaling cascades (DNA damage response, DDR) [10, 29–31]. CPT-11 treatment induced a clearly detectable phosphorylation of ATM, ATR, CHK1, and CHK2. L-OHP evoked phosphorylation of ATM only weakly and we could hardly detect phosphorylation of ATR, CHK1 and CHK2 in L-OHP-treated cells (Figure 2A).

N-terminal phosphorylation of p53 at serine residues S15/S20 by ATM, ATR, CHK1/CHK2, and other kinases stabilizes and activates p53 [31, 32]. Western blot analysis of p53 after treatment with L-OHP and CPT-11 showed that these drugs comparably induced phosphorylation of p53 at S20 in a time-dependent manner. CPT-11 induced phosphorylation at S15, but L-OHP poorly caused phosphorylation of p53 at this site. A roughly equal time-dependent accumulation of p53 occurred with both agents (Supplementary Figure 1A).

DNA damage and replicative stress evoke the phosphorylation of the histone variant H2AX at S139 (γH2AX) by checkpoint kinases [10, 33]. L-OHP induced γH2AX slightly during early (2-6 hours) and later time points of treatment (24 hours). In contrast, CPT-11 induced an immediate, continuing accumulation of γH2AX from 2-24 hours (Figure 2B). We quantified γH2AX with a fluorophore-coupled antibody. Flow cytometry analyses demonstrated that a 3.5-fold accumulation of total cellular γH2AX fluorescence after a 2-hour treatment was increased to 21.5-fold after a 24-hour treatment with CPT-11. A weak, statistically not significant accumulation of γH2AX was noted after L-OHP treatment for 24 hours (Figure 2C). These data are congruent with the unequal activation of checkpoint kinases by L-OHP and CPT-11 (Figure 2A).

Next, we asked whether the accumulation of γH2AX occurs in a cell cycle-specific manner. DNA staining with DAPI confirmed the depletion of S-phase cells after a 24-hour treatment with L-OHP (Figure 2D, compare with Figure 1A-1B). While we detected no significant increase in γH2AX in L-OHP-treated cells, γH2AX-positive cells appeared in the S- and G_2_/M-phases after 6 hours and more pronouncedly in the G_2_/M-phase after 24 hours of CPT-11 treatment (Figure 2D).

These data illustrate that CPT-11 activates the checkpoint kinase signaling cascade strongly and that L-OHP causes a merely transient induction thereof.

### Evaluation of drug-induced cell death of HCT116 cells

**Figure 3:**
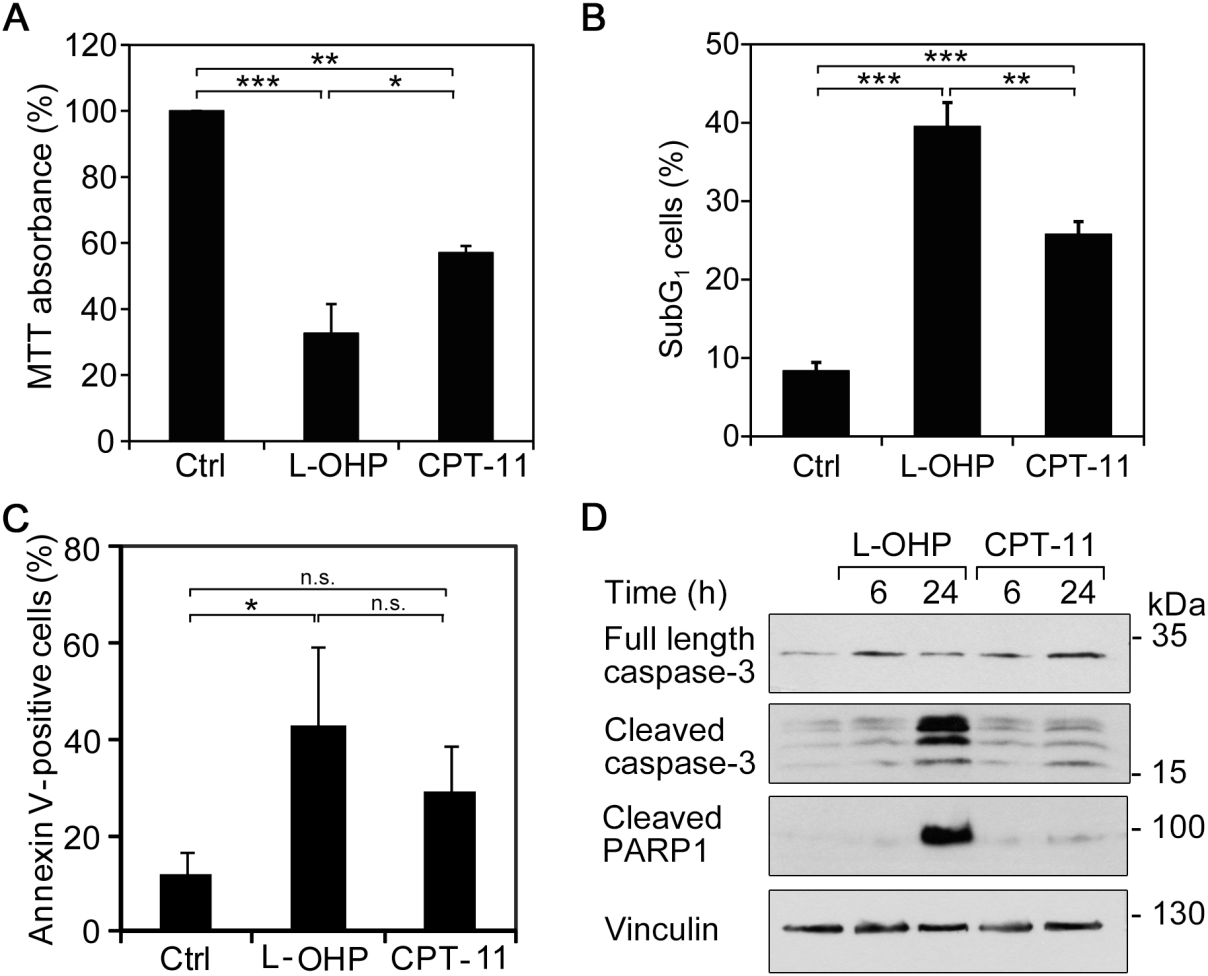
L-OHP and CPT-11 produce different cytotoxic effects Cells were treated with 5 μM L-OHP, 10 μM CPT-11 or DMSO (Ctrl). **(A)** MTT assay measures metabolic activity of cells after 48 hour treatments (n = 3). **(B)** Flow cytometric analysis of subG_1_ cells after 48 hours treatments (n = 3). **(C)** Immunostaining with Annexin V-FITC and flow cytometry detecting apoptotic cells after 48 hour treatments (^*^ p < 0.05, ^**^p < 0.01, ^***^p < 0.001, n = 3). **(D)** Western blot analysis of caspase activation using antibodies against full-length and cleaved caspase-3, as well as cleaved PARP1; vinculin serves as loading control.

To characterize the cytotoxic potential of L-OHP and CPT-11 in HCT116 cells, we used the MTT test. This assay measures the potential of intact cells to reduce the tetrazolium dye MTT from a yellow to a violet substance. MTT activity can therefore serve as read-out for cell viability. L-OHP decreased cell viability to 32.7% and CPT-11 decreased it to 57.0% after 48 hours (Figure 3A).

The MTT assay cannot differentiate between anti-proliferative and cytotoxic effects. Therefore, we determined the percentage of cells in the subG_1_-phase, which we had excluded in previous cell cycle analyses (Figure 1A and 1B). A considerable increase of subG_1_-cells occurred after 48 hours of treatment with either agent. In comparison to 10.4% subG_1_-cells in control cells, L-OHP increased cell death to 37.5%, whereas CPT-11 generated significantly smaller effects with 24.2% (Figure 3B).

The binding of Annexin V to phosphatidylserine residues on the cell surface is a marker for the loss of cell membrane integrity during apoptosis. Untreated HCT116 cell populations contain 14.7% Annexin V-positive cells. L-OHP and CPT-11 increased this fraction to 42.9% and 29.1% after 48 hours, respectively (Figure 3C).

Next, we analyzed apoptotic marker proteins by immunoblot analyses. The executioner caspase-3 is activated by autolytic cleavage and catalyzes the proteolysis and inactivation of the DNA repair enzyme poly-(ADP-ribose)-polymerase 1 (PARP1) [34]. HCT116 cells treated with L-OHP for 6 and 24 hours showed a time-dependent caspase-3 activation and PARP1 cleavage (Figure 3D). A time-dependent accumulation of p53 between 3 and 12 hours preceded the cleavage of PARP1 (Supplementary Figure 1B). In contrast, CPT-11 activated caspase-3 and PARP1 cleavage to a significantly lesser extent (Figure 3D).

We conclude that L-OHP is a more potent inducer of apoptosis than CPT-11.

### L-OHP and CPT-11 regulate pro- and anti-apoptotic factors dissimilarly

**Figure 4:**
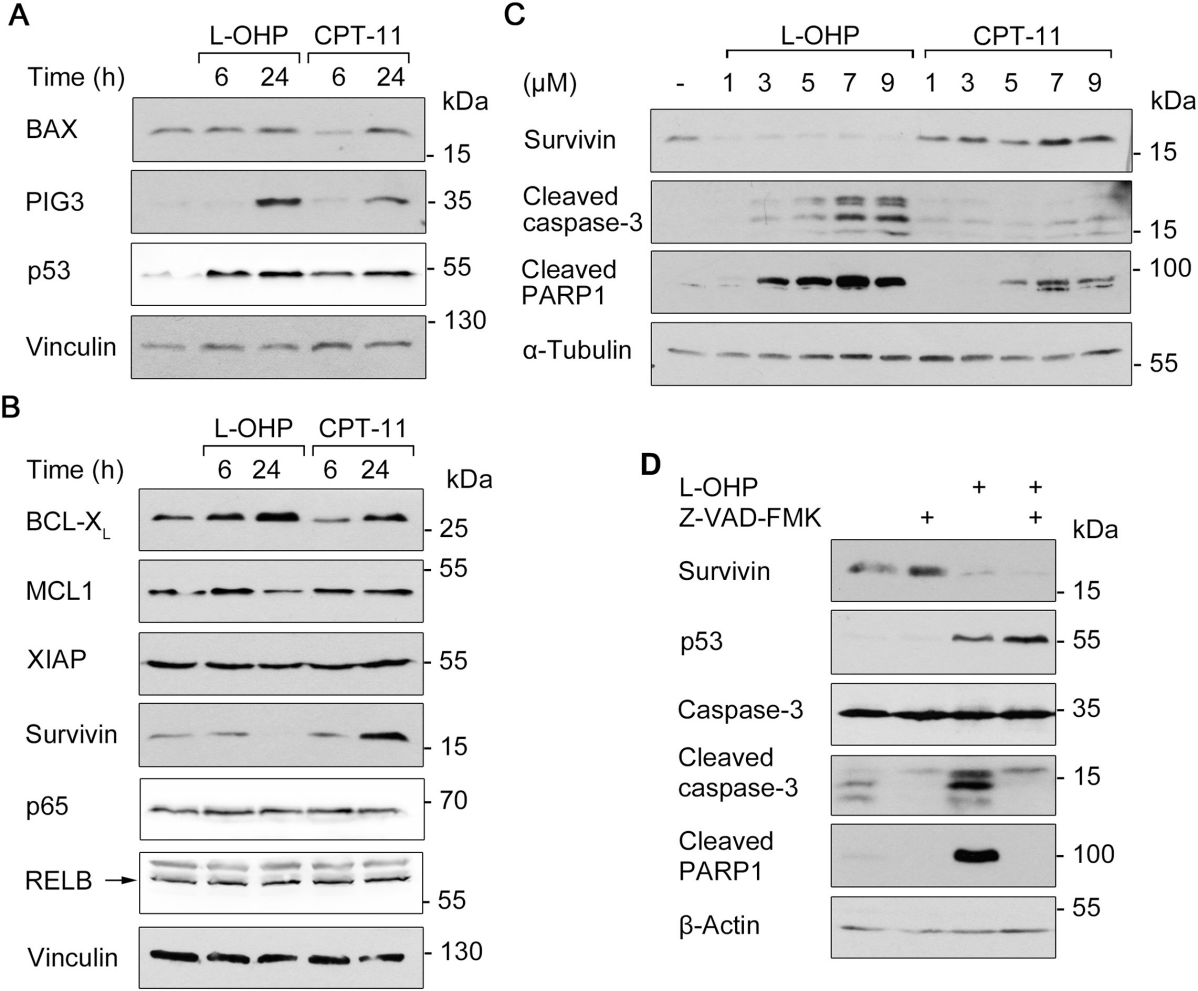
Apoptosis and survival signaling after L-OHP and CPT-11 **(A)** Western blot analysis using antibodies against p53 and pro-apoptotic BAX and PIG-3 after treatment with 5 μM L-OHP or 10 μM CPT-11. **(B)** Immunodetection of NF-κB p65, RELB and anti-apoptotic survivin, XIAP, BCL-X_L_ and MCL1; vinculin serves as loading control. **(C)** Effects of increasing doses L-OHP and CPT-11 on caspase-3 and PARP1 cleavage after 24 hours treatment; α-tubulin serves as loading control. **(D)** Cells were treated with a combination of L-OHP and the caspase-inhibitor Z-VAD-FMK (50 μM). Immunodetection of survivin, p53 and full-length caspase-3 was conducted. Detection of apoptosis was determined by cleavage products of caspase-3 and PARP1; β-actin serves as loading control. Please note: Figure 4A and 4B, as well as Supplementary Figure 2A show signals acquired by different detection methods, but originate from the same Western blots. This is due to a switch in the immunoblot chemiluminescence detection system from X-ray films (darker background) to a CCD camera system (Fusion Solo S, Vilber Lourmat; lighter background).

We analyzed the levels of pro- (Figure 4A) and anti-apoptotic factors (Figure 4B) to determine mechanisms by which L-OHP and CPT-11 kill HCT116 cells. BCL2-associated X protein (BAX) and p53-inducible gene 3 (PIG3) are pro-apoptotic transcriptional targets of p53 [10, 31, 32]. Western blot showed that treatment with L-OHP and CPT-11 for 24 hours induced the expression of PIG3, but not of BAX. Accumulation of p53 was comparable after both treatments (Figure 4A; congruent with Supplementary Figure 1A). An increased expression of the anti-apoptotic NF-κB target gene BCL2 family member B-cell lymphoma extra-large (BCL-X_L_) was detectable after L-OHP and CPT-11 treatment. The BCL family protein myeloid cell leukemia 1 (MCL1) and XIAP were unaffected by both treatments. Protein levels of the NF-κB family members p65 and RELB did also not change. We though noted a strikingly divergent regulation of survivin. After 24 hours, CPT-11 induced and L-OHP downregulated the levels of survivin (Figure 4B).

This finding prompted us to analyze the regulation and functions of survivin further. Time-course analyses revealed that 5 μM L-OHP led to an accumulation of p53 after 6 to 12 hours and this correlated with a decrease of survivin. PARP1 cleavage occurred concurrently with the loss of survivin (Supplementary Figure 1B). When we treated HCT116 cells with increasing doses of L-OHP and CPT-11 for 24 hours, we found that 1 μM of L-OHP sufficed to suppress survivin and that doses at and higher than 3 μM induced apoptosis. Up to 7 μM CPT-11 induced survivin levels and activated caspase-3 and the cleavage of PARP1 weaker than equimolar doses of L-OHP did (Figure 4C).

We suspected that caspases cleave survivin during L-OHP-induced apoptosis. However, the pan-caspase inhibitor Z-VAD-FMK did not rescue survivin in the presence of L-OHP (Figure 4D).

Next, we investigated whether genotoxic insults of L-OHP or the cell cycle effects determine survivin expression in HCT116 cells. We arrested them with a double-thymidine block in the early S-phase and analyzed survivin protein levels as well as cell cycle progression for up to 12 hours post release from the cell cycle block (Supplementary Figure 2A and 2B). When the major portion of HCT116 cells arrested in the early S-phase, the lowest survivin levels were detected (0 and 2 hours post thymidine block). Cells progressed to S-phase 2 hours after release. During 4-8 hours after release, when they entered S-phase and G_2_/M-phase, survivin protein levels increased markedly. At 10 and 12 hours after release, the majority of cells entered G_1_-phase again and during this time, survivin protein amounts decreased (Supplementary Figure 2A and 2B). This fluctuation of survivin expression was not associated with any change in γH2AX levels (Supplementary Figure 2A). These data are coherent with the repression of survivin by L-OHP despite no significant accumulation of γH2AX (Figure 2B-2D).

To sum up, our data show a drug- and cell cycle-dependent expression of survivin.

### Expression of survivin determines apoptosis induction after L-OHP and CPT-11 treatment

Our results suggest that the increased or decreased levels of survivin determine the cytotoxic potential of CPT-11 and L-OHP. If this is the case, a reduction of survivin should increase the pro-apoptotic potential of CPT-11 and an overexpression of survivin should attenuate the pro-apoptotic effects of L-OHP.

**Figure 5:**
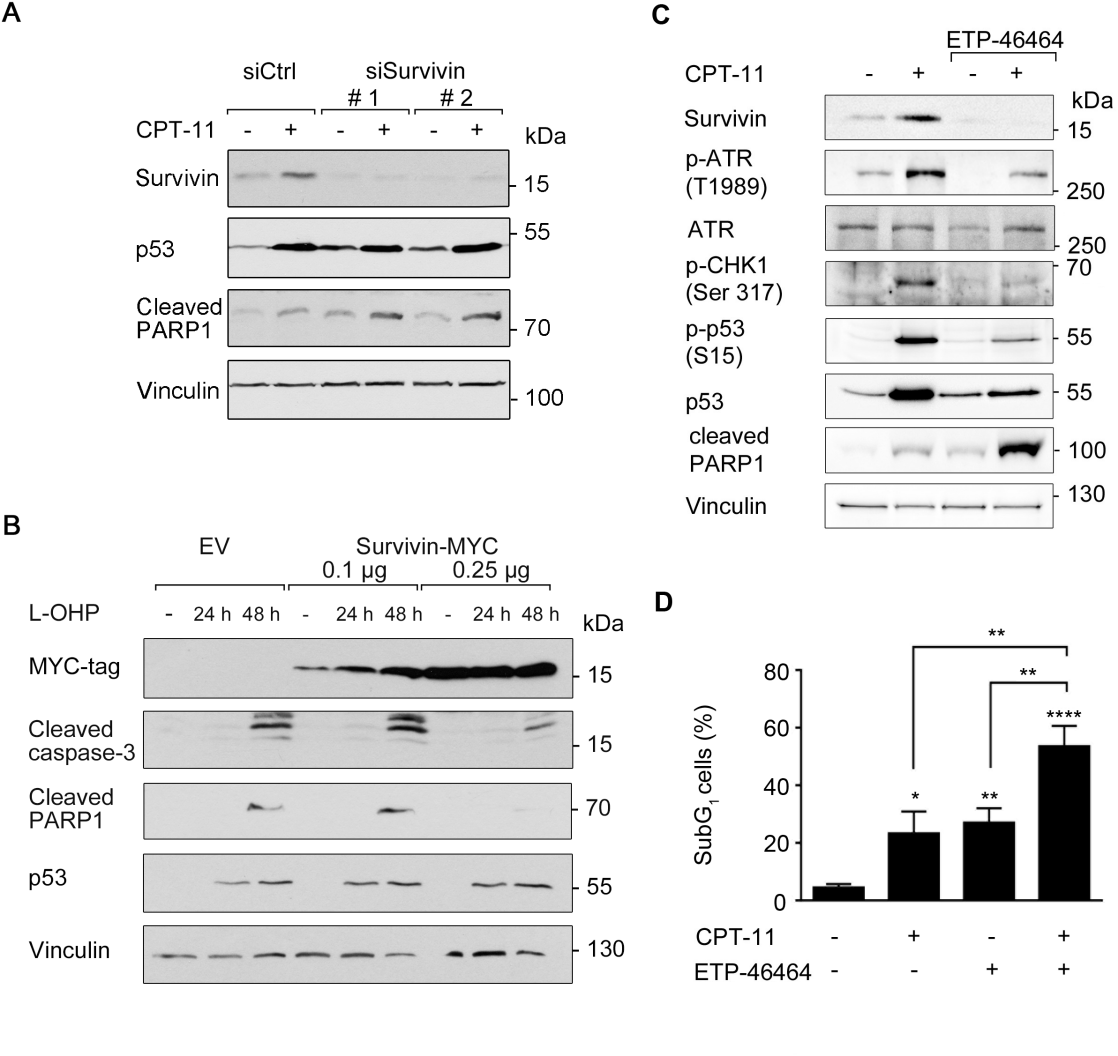
Survivin affects cellular susceptibility to chemotherapeutic drugs **(A)** siRNA-mediated knockdown of survivin was performed in HCT116 cells for 24 hours (scrambled siRNA (siCtrl) transfection serves as control). Thereafter, cells were treated with 10 μM CPT-11 for 24 hours. Western blot analysis detected protein levels of survivin, p53, as well as cleavage products of caspase-3 and PARP1; vinculin serves as loading control. **(B)** HCT116 cells were transfected with 0.1 μg and 0.25 μg survivin-MYC plasmid for 24 hours and were treated 5 μM L-OHP for additional 24 and 48 hours. Western blot analysis detected MYC-tag, cleavage of caspase-3 and PARP1; vinculin serves as loading control (n = 2). **(C)** HCT116 cells were treated with 3 μM ETP-46464 for 1 hour, after which 10 μM CPT-11 were added for additional 24 hours. Western blot was carried out as indicated, with vinculin as loading control (n = 2). **(D)** HCT116 cells were treated as described in C, but for 48 hours total incubation time. Cells were harvested and analyzed for the occurrence of cells in the subG1 fraction (n=3).

To evaluate such presumed effects of survivin on chemotherapy-induced apoptosis, we performed a knockdown of survivin. Two independent siRNA oligomers (siSurvivin #1 and #2) suppressed survivin protein levels significantly, but not the accumulation of p53 (Figure 5A). Indeed, CPT-11 increased the caspase-mediated, apoptotic PARP1 cleavage more pronouncedly in cells with decreased levels of survivin (Figure 5A).

Next, we transfected HCT116 cells with increasing amounts of an overexpression construct encoding MYC-tagged survivin. After 24 hours, we treated the cells with L-OHP for 24 to 48 hours. L-OHP-treated empty vector (EV)-transfected cells activated caspase-3 and showed a cleavage of PARP1. Overexpression of survivin attenuated the L-OHP-induced activation of caspase-3, but not the accumulation of p53 (Figure 5B).

Since we observed that CPT-11 triggered the ATR-CHK1 axis and an accumulation of survivin (Figures 2A, 4B, 5A and Supplementary Figure 1B), we tested whether these processes are functionally connected and provide a potential option to kill colon cancer cells. To impair the ATR-CHK1 axis, we used the ATR inhibitor (ATRi) ETP-46464 [35]. As expected, ETP-46464 suppressed the CPT-11-induced phosphorylation of ATR and its downstream target CHK1 as well as the accumulation of p53 in HCT116 cells (Figure 5C).

Additionally, treatment with CPT-11 and ETP-46464 reduced the accumulation of survivin strongly and increased the cleavage of PARP1, which is a marker for apoptosis (Figure 5C). Analysis of DNA fragmentation by flow cytometry verified that the combination of CPT-11 and ETP-46464 was significantly more pro-apoptotic than the individual application of either agent (54% versus 23%-27%; Figure 5D).

To exclude that these observations are limited to CPT-11, we used hydroxyurea as additional inducer of replicative stress and survivin [13, 36–38]. Inhibition of ATR with ETP-46464 also reduced the hydroxyurea-induced accumulation of survivin and enhanced apoptosis (Supplementary Figure 3A-3C).

We conclude that the L-OHP-mediated suppression of survivin can explain why L-OHP induces apoptosis more effectively than CPT-11.

### Transcriptional suppression of survivin by L-OHP depends on p53

**Figure 6:**
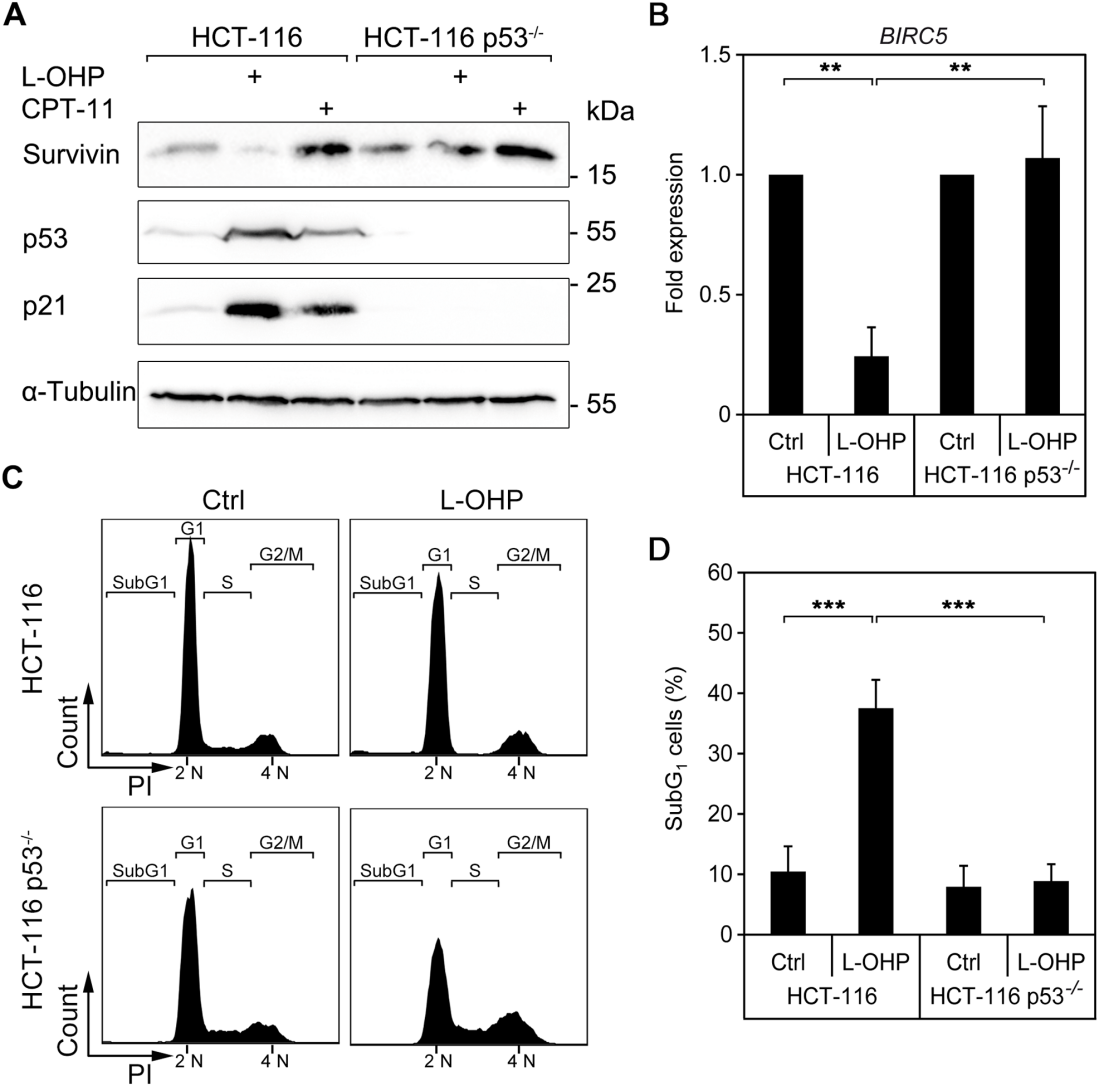
Induction of cell death and suppression of survivin after L-OHP depends on p53 **(A)** HCT116 wild typeand p53^-/-^ cells were treated with 5 μM L-OHP or 10 μM CPT-11 for 24 hours. Protein levels of survivin, p53 and p21 were detected by Western blot analysis; vinculin serves as loading control. **(B)** Quantitative real-time PCR was performed to quantify *BIRC5* mRNA levels in HCT116 wild type and p53-deficient cells after 24 hours treatment (^**^ p < 0.01, n = 3). **(C)** Flow cytometric analysis of DNA content was done in HCT116 wild type and p53^-/-^ cells after 24 hours treatment with L-OHP (n = 4). **(D)** SubG_1_-populations were detected in both cell lines after 48 hours treatment (^***^ p < 0.001, n = 4).

Since p53 is an essential regulator of chemotherapeutic sensitivity [31, 32, 37, 39, 40], we investigated whether p53 regulates the modulation of survivin by L-OHP and CPT-11. We treated HCT116 wild type and p53-deficient cells with these drugs. As reported [37], compared to p53-proficient cells, p53-deficient cells express higher levels of survivin. L-OHP did not suppress survivin in p53^-/-^ cells after 24 hours, while the CPT-11-mediated accumulation of survivin remained unaffected in both cell lines (Figure 6A). Quantitative real time PCR revealed a nearly fivefold, statistically significant reduction of the *BIRC5* mRNA in L-OHP-treated p53-positive HCT116 cells (Figure 6B). This finding suggests that L-OHP represses survivin by a p53-dependent transcriptional mechanism.

To test if other p53-negative colon cancer cells also fail to repress survivin, we treated three short-term cultured colon cancer cell lines (HROBMC01, HROC43, HROC239) with L-OHP. As in p53-deficient HCT116 cells, L-OHP could not suppress survivin expression in these cell lines (Supplementary Figure 4).

While L-OHP stalled cell cycle progression of p53-proficient HCT116 cells (G_1_: 69.4 ± 7.9%, S: 6.0 ± 4.7%, G_2_/M: 24.5 ± 7.3%), p53-deficient cells did not build up this G_1_ cell cycle checkpoint and continued to enter S-phase (G_1_: 54.6 ± 9.9%, S: 17.4 ± 11.8%, G_2_/M: 28.0 ± 3.7%) (Figure 6C). This lack of cell cycle arrest is associated with a rescue of *BIRC5* gene expression in p53-deficient cells and no accumulation of p21 (Figure 6A and 6B). Coherent with the cytoprotective role of survivin in cells exposed to L-OHP (Figure 5B), the measurement of subG_1_ fractions indicated that L-OHP was not toxic for p53^-/-^ HCT116 cells (Figure 6D).

Hence, p53 is required to suppress survivin and to induce apoptosis in HCT116 cells exposed to L-OHP.

### The p53 target gene p21 controls the expression of survivin

**Figure 7:**
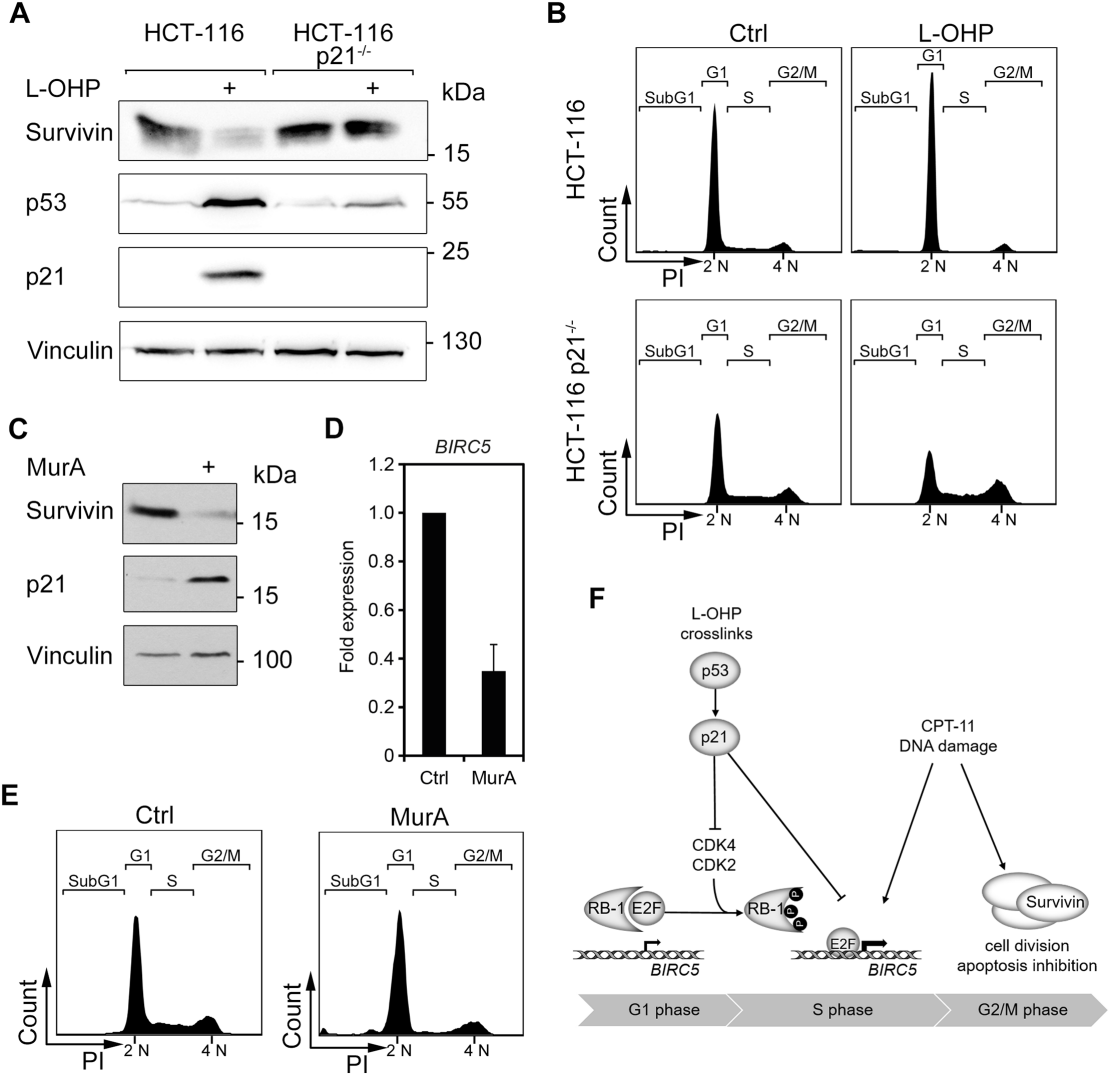
The p53-p21 axis facilitates downregulation of survivin via cell cycle **(A)** HCT116 wild typeand p21-deficient (p21^-/-^) cells were treated with 5 μM L-OHP for 24 hours. Whole cell lysates were analyzed with antibodies against p53, p21, and survivin; vinculin serves as loading control. **(B)** Cell cycle distribution was analyzed after 24 hours treatment by flow cytometry analysis (n = 3). **(C)** To induce p21, RKO p21^ind^ cells were treated with 3 nM Muristerone A for 24 hours and tested for the levels of p21 and survivin; vinculin, loading control. **(D)** *BIRC5* mRNA levels were analyzed by quantitative real-time PCR after 24 hours treatment with MurA in RKO p21^ind^ cells (n = 3). **(E)** Cell cycle distribution was measured by flow cytometry analyses of cellular DNA content (n = 3). **(F)** Scheme summarizing the supposed mechanisms of survivin regulation after L-OHP and CPT-11 treatment.

Next, we asked whether the L-OHP-mediated suppression of survivin relies on p53-mediated cell cycle effects or whether p53 exerts a direct suppressive function. As a p53-dependent expression of the cell cycle regulator p21 arrest cells in G_1_-phase, we elucidated whether p21 controls survivin expression in HCT116 cells and otherwise isogenic p21-deficient HCT116 cells. We found that L-OHP did not reduce survivin in HCT116 p21^-/-^ cells (Figure 7A). Moreover, L-OHP-treated p21^-/-^ cells did not arrest in G_1_ and continued to enter the S-phase (Figure 7B). We though noted low p53 protein levels in HCT116 p21^-/-^ cells (Figure 7A), presumably due to a loss of the positive feedback signaling between p21 and p53 [41].

To extend these data, we overexpressed p21 in genetically engineered human RKO colorectal cancer cells (RKO p21^ind^; Figure 7C-7E). Such cells possess a stably transfected p21 expression plasmid, which can be induced with the phytoecdysteroid analog Muristerone A (MurA) [42]. We observed that the overexpression of p21 was sufficient to reduce the protein and the mRNA levels of survivin (Figure 7C and 7D). As anticipated, the induction of p21 halted cells in G_1_ and depleted the S-phase population (Figure 7E).

We conclude that a p21-mediated cell cycle arrest in the G_1_-phase can suppress survivin expression.

## DISCUSSION

The identification of marker proteins that indicate the success of chemotherapy is of outstanding clinical relevance. Moreover, such factors are a key to personalized medicine [43]. Survivin is a prognostic marker that indicates poor therapeutic success in colorectal cancer, non-small cell lung carcinoma, and other tumors [22, 44–46]. We report that L-OHP downregulates survivin and that CPT-11 induces survivin. Furthermore, we demonstrate that a knockdown of survivin increases the cytotoxicity of CPT-11 and that the overexpression of survivin in L-OHP-treated cells is cytoprotective.

We were particularly interested in this divergent control of survivin by chemotherapeutics, because of its essential roles in cellular stemness and robustness. Survivin ensures proper formation of the chromosomal passenger complex during mitosis, to prevent aneuploidy and to ensure chromosomal stability [25, 26, 47, 48]. Moreover, cytoplasmic survivin interacts with the X-linked inhibitor of apoptosis (XIAP) to inhibit caspases-3, -7, and -9, which catalyze the demise of cellular proteins during apoptosis [ 24- 26, 47, 48]. Accordingly, survivin is overexpressed in various malignant tumors and cancer stem cells [24–26], and elevated survivin levels indicate poor responses to chemo-/radiotherapy and drug resistance. Therefore, survivin is an appreciated therapeutic target [26, 47, 49]. We demonstrate that a reduction of CPT-11-induced survivin enhances apoptotic effects, which warrants further investigations on a chemosensitizing effect of survivin antagonists.

The modulation of cell cycle progression by L-OHP and CPT-11 can largely explain their divergent effects on survivin. CPT-11 inhibits topoisomerase I and consequently stalls cells in the late S- to G_2_/M-phase. L-OHP crosslinks DNA and stalls cell cycle progression by inhibition of DNA replication and transcription. L-OHP significantly induces p53 and its downstream target p21 and thereby causes a cell cycle arrest in the G_1_-phase. We further demonstrate that L-OHP influences survivin levels through p53 and p21. From these findings and our cell cycle release experiments, we conclude that stalled cell cycle progression suppresses *BIRC5* expression after DNA crosslinking. Congruently, cancer cell lines lacking p53 or p21 do not undergo a cell cycle arrest in the G_1_-phase and survivin remains expressed in response to L-OHP. Hence, the p53-p21 axis is indispensable for the transcriptional repression of survivin after L-OHP treatment. This finding supports previous publications showing that the p53-p21 pathway is essential for L-OHP-mediated cytotoxicity [50, 51]. In contrast, p53 is not critical for the cytotoxicity of CPT-11, which activates p53 and p21, but does not suppress *BIRC5* expression (Figure 7F).

CPT-11 leads to an accumulation of cells in the G_2_/M-phase, E2F activity remains elevated despite an increase in p21, and survivin accumulates. These findings are consistent with divergent types of cell cycle arrest in response to L-OHP and CPT-11. Since the overexpression of p21 alone decreases *BIRC5* gene expression and prevents an accumulation of survivin after treatment with CPT-11, we deduce that the different effects of L-OHP and CPT-11 on cell cycle progression determine survivin expression, and ultimately, apoptosis. The *BIRC5* gene is regulated in a cell cycle-dependent manner by the transcription factors E2F1-3 and SP1/SP3 [26, 52]. RB1 binds to the *BIRC5* promoter to block E2F-dependent transcription of *BIRC5* [52]. Several sequential phosphorylation events inactivate RB1. In complexes with D-type cyclins, the cyclin-dependent kinase-4 (CDK4) phosphorylates and inactivates RB1 [27, 28]. This liberates E2F and allows E2F-dependent gene expression promoting G_1_-phase-to-S-phase transition and the expression of survivin. CDK2/cyclin E and CDK1/cyclin B complexes catalyze the phosphorylation of RB1 from S-phase to mitosis. PP1 and PP2A phosphatase complexes dephosphorylate RB1 when the daughter cells exit M-phase [27, 28]. Accordingly, survivin increases during cell cycle progression to M-phase and drops upon G_1_-phase re-entry [26]. p21 inhibits CDK4/Cyclin D, CDK2/Cyclin B complexes and the proliferating cell nuclear antigen (PCNA) which are required for S-phase progression [10, 31, 32]. Consistent with these data, we find that CPT-11 leads to a hyperphosphorylation of RB1 and increased E2F-activity and L-OHP suppresses E2F-activity and RB1 levels (Figure 7F).

Survivin also belongs to a group of proteins that the transcription factors p53 and NF-κB regulate ambivalently. While p53 and p21 suppress survivin in resting cells, drug-induced replicative stress and DNA damage activate survivin and further NF-κB-activated genes dependent on p53 and NF-κB p65 [39]. This is surprising given that p53-mediated target gene activation is associated with cell cycle arrest and apoptosis induction during chemotherapy, while several NF-κB-dependent factors promotes survival effects. However, such a chemotherapy-induced increase of survivin is consistent with the concomitant activation of p53 and NF-κB in various tumors [37, 39, 40, 53]. Nonetheless, we observe a marked increase of survivin in CPT-11-treated, G_2_/M-arrested cells, but no significant upregulation of *BIRC5* mRNA after 6 or 24 hours (data not shown). This finding suggests that CPT-11 augments the protein stability of survivin due to an arrest in the late S- and G_2_/M-phases. This idea is consistent with the literature, which reports that a phosphorylation of survivin at T34 by CDC2/CDK1 increases its protein stability [54]. It is additionally possible that NF-κB sustains *BIRC5* gene expression during stress. Topoisomerase I poisons activate NF-κB [7, 8], and replicative stress triggers crosstalk between NF-κB/p53 and an induction of survivin [37, 39, 40, 53]. In contrast, crosslinking substances (e.g., platinum agents like L-OHP) activate NF-κB poorly [55].

It has been reported that γH2AX accumulates L-OHP-exposed cells lacking p53 [56] and a study comparing p53-positive and p53-negative HCT116 cells reported increased L-OHP-induced DNA damage in HCT116 cells lacking p53 [57]. In agreement, γH2AX was hardly apparent in p53-positive HCT116 cells. Thus, the repression of survivin in response to L-OHP and its induction by CPT-11 cannot be explained by an increased DNA damage induction by L-OHP. Therefore, we conclude that the divergent accumulation of γH2AX-positive cells in CPT-11- and L-OHP-treated HCT116 cells is not correlated with DNA damage and apoptosis. The meek phosphorylation of p-H2AX in L-OHP-treated HCT116 cells rather suggests a rapid removal of platinum adducts from DNA by the NER pathway, which is modulated by p53 [4, 33]. In line with this hypothesis, the activation of caspase-3 in L-OHP-treated HCT116 cells is a marker for the recognition of such adducts by GG-NER. It should be considered that GG-NER can remove platinum-induced ICLs from DNA, but that HR will not be executed in L-OHP-treated G_1_-phase-arrested cells due to the lack of an intact sister strand [10, 31, 58]. Since TC-NER and translesion polymerases repair L-OHP-induced ICLs in a DNA replication-independent manner [3, 5], we assume that this pathway removes platinum-DNA adducts in L-OHP-treated, non-cycling HCT116 cells.

Consistent with the poor increase of γH2AX, L-OHP hardly induces checkpoint kinase signaling. Apparently, the arrest of cells and a minor number of cells passing S-phase prevents a strong activation of ATM, ATR, CHK1, and CHK2 after L-OHP treatment. These data are consistent with the proliferation-dependent activation of these checkpoint kinases in HCT116 cells treated with the heterocyclic aromatic amine PhIP, which generates bulky DNA lesions [29]. Hence, checkpoint kinase activation and the accumulation of γH2AX are not linked to the suppression of survivin and the induction of apoptosis in response to L-OHP. Further support for a DNA damage-independent attenuation of survivin by L-OHP comes from cell cycle release experiments. These show that survivin levels fluctuate dependent on the cell cycle under conditions of no DNA damage.

Despite the comparably low levels of L-OHP-induced checkpoint kinase activation, we observed phosphorylation of p53 at S20. These low checkpoint kinase activation levels might be sufficient to catalyze phosphorylation of p53 at S20 and/or that other kinases [10, 31, 32] phosphorylate p53 in response to L-OHP. Apparently, this phosphorylation can stabilize p53 to induce its positively regulated targets PIG3 and p21 as well as to suppress its negatively regulated target survivin. Further analyses are necessary to identify the L-OHP-activated kinase for the phosphorylation of p53 at S20.

Our preclinical data may suggest an option to stratify colon cancer patients according to their tumor-associated p53, p21, and survivin levels to therapies containing L-OHP- or CPT-11. Since the activation of ATM-CHK2 and ATR-CHK1 supports DNA repair and survival processes in CPT-11-treated colon cancer cells [7, 16–19], a combination of CPT-11 with inhibitors of these kinases could be a therapeutic option. Indeed, CPT-11-induced survivin is affected by an ATRi and this is associated with increased colon cancer cell death. Our data additionally verify that a pharmacological inhibition of ATR blocks both the CPT-11-induced phosphorylation of CHK1 and the accumulation of p53. This finding is important in light of the fact that a novel inhibitor of CHK1 could accentuate anti-tumor effects of CPT-11 against p53-negative human colon cancer xenografts in mice without additional undesired toxicity to healthy tissue [59].

In sum, we provide evidence that a differential regulation of survivin determines the efficiency of CPT-11 and L-OHP against colorectal cancer cells. Ablation of survivin is a major mechanism through which L-OHP induces apoptosis. These results define pro-apoptotic mechanisms of crosslinking agents better. A combination of CPT-11 with RNAi against survivin and an ATRi improves the cytotoxicity of CPT-11. This finding might be translated into clinical applications.

## MATERIALS AND METHODS

### Cell cultivation, treatment, and transfections

Cells were cultured in Dulbecco’s Modified Eagles Medium containing 4.5 g/l glucose (Sigma-Aldrich, USA) and 10% fetal calf serum (PAA laboratories, Austria) to a maximum of 30 passages. Absence of *Mycoplasma* infections was tested with the MycoAlert™ kit (Lonza, Switzerland) every 4-8 weeks. HCT116 wild type, p53^-/-^ and p21^-/-^ cells were obtained from Prof. Dr. B. Vogelstein (Johns Hopkins University, Baltimore, USA). Authentication of HCT116 and RKO cells cells was done by DNA fingerprint at the Leibniz Institute, DSMZ GmbH, (Braunschweig, Germany). RKO p21^ind^ cells were a gift from Prof. Dr. W. Wels (Georg-Speyer-Haus, Frankfurt/Main, Germany). These were treated with 3 nM Muristerone A (MurA, Alexis Biochemicals, USA) for 24 hours to induce p21; details on these cells are explained [42]. HROC43, HROC239 T0 M1 and HROBMC01 were generated and cultured as described [60, 61]. Early passages below 40 were used. Cells were treated with L-OHP, CPT-11 (Selleckchem, USA, dissolved in DMSO), hydroxyurea (Sigma-Aldrich, Germany, dissolved freshly in ddH2O) and ETP-46464 (Cayman, USA, dissolved in DMSO) as indicated. Control treatment was done with equal amounts of DMSO. Transfections of plasmids and siRNA were done with lipofectamine^®^2000 (Thermo Fisher Scientific, USA) according to the manufacturer´s protocol. SiRNA sequences: *BIRC5#1* fwd 5’-*UAGAUGUUUCAACUGUGCUCUUGUU*-3‘, *BIRC5#1* rev 5’-*AACAAGAGCACAGUUGAAACAUCUA*-3‘, *BIRC5#2* fwd 5’-*AACAACAUGAGGUCCAGACACAUUC*-3‘, *BIRC5#2* rev 5’-*GAAUGUGUCUGGACCUCAUGUUGUU*-3‘.

### Double-thymidine block

Cells were treated with 2 mM thymidine (Sigma-Aldrich) for 18 hours and released for 9 hours in fresh growth medium. This was followed by a second thymidine treatment for 18 hours and a release in thymidine-free medium. Cells were harvested and analyzed by immunodetection and flow cytometry.

### Whole cell lysis, SDS page, and immunoblot analysis

Cells were harvested and lysed in NaCl-EDTA-Tris-Nonidet (NETN) buffer containing proteinase inhibitor cocktail, 1 mM sodium-orthovanadate (Na_3_OV_4_, Sigma-Aldrich) and 5 mM sodium fluoride (NaF, Sigma-Aldrich). SDS page and immunoblot are summarized in [13, 36, 53]. Immunoblots are representative for minimum three independently repeated experiments, if not stated differently. Please note that a subset of immunoblot signals was acquired by different detection methods. This is due to a switch in the immunoblot chemiluminescence detection system from X-ray films (darker background) to a CCD camera system (Fusion Solo S, Vilber Lourmat; lighter background; Figure 4A, 4B and Supplementary Figure 2A). Furthermore, blots shown in Supplementary Figure 4 were detected with the Odyssey InfraRed system [13, 62]. Antibodies were from Santa Cruz Biotechnology, USA: BAX #sc-20067, PIG3 #sc-30068, p53 #sc-81168, p21 #sc-6246, caspase-3 #sc-7272, ATR (ph-S428) #sc-2853, CHK1 #sc-8408, MCL1 #sc-819; Sigma-Aldrich: β-actin #A-2066, α-tubulin #T5168; Abcam, UK: ATM #ab32420, ATM (ph-S1981) #ab81292, CHK2 (ph-T68) #ab32148; Bethyl Laboratories, USA: CHK1 (ph-S317) #A300-163A; Cell Signaling Technology, USA: ATR #2790, RB1 (ph-S780) #9307, cleaved caspase-3 #9664, p53 (ph-S15) #9284, p53 (ph-S20) #9287; Aviva Systems Biology, USA: RB1 #ARP58065, Cyclin B2 #ARP63411; Biozol, Germany: Vinculin #BZL-03106; Merck Millipore, Germany: γH2AX (S139) #05-636; BD Pharmingen, USA: BCLXL #551022, cleaved PARP1 #552596, XIAP #610716; Novus Biologicals, USA: Survivin #NB500-201.

### Quantitative real-time PCR

Cellular mRNA was isolated by trizol extraction with peqGOLD RNAPure™ (PeqLab, Erlangen, Germany) according to the manufacturer, followed by reverse transcription using the RevertAid First Strand cDNA Synthesis Kit and Oligo(dT)_18_ primers (Thermo Fisher Scientific). Real-time PCR was conducted using Power SYBR Green PCR Master Mix (Applied Biosystems, USA). Data were analyzed with the ΔC_q_ quantification model [53], using two reference genes (HMBS, GAPDH). Primer sequences for qPCR: *BIRC5* fwd 5′-*GACGACCCCATAGAGGAACA*-3‘, *BIRC5* rev 5′-*CCATGGCAGCCAGCTGCTCG*-3‘, *GAPDH* fwd 5′-*TGCACCACCAACTGCTTAGC*-3‘, *GAPDH* rev 5′-*GGCATGGACTGTGGTCATGAG*-3‘, *HMBS* fwd 5′-*GGCAATGCGGCTGCAA*-3‘, *HMBS* rev 5′-*GGGTACCCACGCGAATCAC*-3‘.

### Cell viability assay (MTT) and luciferase assay

These assays were performed as described [37], with the pE2F-TA-Luc plasmid (Clontech Laboratories, USA).

### Flow cytometry analysis

Cell fixation and staining of DNA content with propidium iodide (PI) was done as described [13, 36, 53]. Living cell populations were gated by excluding subG_1_-fractions. Staining of apoptotic cells with Annexin V-APC antibody was performed as in [39]. Cytometric assessment of histone H2AX phosphorylation was done with FITC-coupled antibody against γH2AX (ph-S139, Merck Millipore: #16-202A) [63]. For staining of DNA content, DAPI was added to the cells shortly before measurement. Flow cytometry was conducted with a BD FACS Canto™ II (Beckton Dickinson, USA). Total fluorescence intensity was determined by area-under-the-curve-calculation. Evaluation of cytometry data was done with the FlowJo7.6.5 software.

### Statistical analysis

Graphs show mean and standard deviation out of independent experiments. The significance of differences between experimental conditions was determined using one-way ANOVA and post-hoc Bonferroni´s multiple comparison test out of three independent experiments minimum.

## SUPPLEMENTARY MATERIALS FIGURES



## References

[1] Haggar FA, Boushey RP (2009). Colorectal cancer epidemiology: incidence, mortality, survival, and risk factors. Clin Colon Rectal Surg.

[2] Carrato A (2008). Adjuvant treatment of colorectal cancer. Gastrointest Cancer Res.

[3] Bowden NA (2014). Nucleotide excision repair: why is it not used to predict response to platinum-based chemotherapy?. Cancer Lett.

[4] Marteijn JA, Lans H, Vermeulen W, Hoeijmakers JH (2014). Understanding nucleotide excision repair and its roles in cancer and ageing. Nat Rev Mol Cell Biol.

[5] Enoiu M, Jiricny J, Scharer OD (2012). Repair of cisplatin-induced DNA interstrand crosslinks by a replication-independent pathway involving transcription-coupled repair and translesion synthesis. Nucleic Acids Res.

[6] Nikolova T, Kiweler N, Krämer OH (2017). Interstrand Crosslink Repair as a Target for HDAC Inhibition. Trends Pharmacol Sci.

[7] Pommier Y (2006). Topoisomerase I inhibitors: camptothecins and beyond. Nat Rev Cancer.

[8] Xu Y, Her C (2015). Inhibition of Topoisomerase (DNA) I (TOP1): DNA Damage Repair and Anticancer Therapy. Biomolecules.

[9] Liu S, Shiotani B, Lahiri M, Marechal A, Tse A, Leung CC, Glover JN, Yang XH, Zou L (2011). ATR autophosphorylation as a molecular switch for checkpoint activation. Mol Cell.

[10] Dobbelstein M, Sørensen CS (2015). Exploiting replicative stress to treat cancer. Nat Rev Drug Discov.

[11] Kurose A, Tanaka T, Huang X, Halicka HD, Traganos F, Dai W, Darzynkiewicz Z (2005). Assessment of ATM phosphorylation on Ser-1981 induced by DNA topoisomerase I and II inhibitors in relation to Ser-139-histone H2AX phosphorylation, cell cycle phase, and apoptosis. Cytometry A.

[12] Kozlov SV, Graham ME, Jakob B, Tobias F, Kijas AW, Tanuji M, Chen P, Robinson PJ, Taucher-Scholz G, Suzuki K, So S, Chen D, Lavin MF (2011). Autophosphorylation and ATM activation: additional sites add to the complexity. J Biol Chem.

[13] Göder A, Emmerich C, Nikolova T, Kiweler N, Schreiber M, Kühl T, Imhof D, Christmann M, Heinzel T, Schneider G, Krämer OH (2018). HDAC1 and HDAC2 integrate checkpoint kinase phosphorylation and cell fate through the phosphatase-2A subunit PR130. Nat Commun.

[14] Okita N, Minato S, Ohmi E, Tanuma S, Higami Y (2012). DNA damage-induced CHK1 autophosphorylation at Ser296 is regulated by an intramolecular mechanism. FEBS Lett.

[15] Ward IM, Wu X, Chen J (2001). Threonine 68 of Chk2 is phosphorylated at sites of DNA strand breaks. J Biol Chem.

[16] Flatten K, Dai NT, Vroman BT, Loegering D, Erlichman C, Karnitz LM, Kaufmann SH (2005). The role of checkpoint kinase 1 in sensitivity to topoisomerase I poisons. J Biol Chem.

[17] Josse R, Martin SE, Guha R, Ormanoglu P, Pfister TD, Reaper PM, Barnes CS, Jones J, Charlton P, Pollard JR, Morris J, Doroshow JH, Pommier Y (2014). ATR inhibitors VE-821 and VX-970 sensitize cancer cells to topoisomerase i inhibitors by disabling DNA replication initiation and fork elongation responses. Cancer Res.

[18] Sakasai R, Teraoka H, Takagi M, Tibbetts RS (2010). Transcription-dependent activation of ataxia telangiectasia mutated prevents DNA-dependent protein kinase-mediated cell death in response to topoisomerase I poison. J Biol Chem.

[19] Zhou Y, Wan G, Spizzo R, Ivan C, Mathur R, Hu X, Ye X, Lu J, Fan F, Xia L, Calin GA, Ellis LM, Lu X (2014). miR-203 induces oxaliplatin resistance in colorectal cancer cells by negatively regulating ATM kinase. Mol Oncol.

[20] Lewis KA, Lilly KK, Reynolds EA, Sullivan WP, Kaufmann SH, Cliby WA (2009). Ataxia telangiectasia and rad3-related kinase contributes to cell cycle arrest and survival after cisplatin but not oxaliplatin. Mol Cancer Ther.

[21] Zhang L, Yu J (2013). Role of apoptosis in colon cancer biology, therapy, and prevention. Curr Colorectal Cancer Rep.

[22] Zheng HC (2017). The molecular mechanisms of chemoresistance in cancers. Oncotarget.

[23] Shen H, Yang J, Huang Q, Jiang MJ, Tan YN, Fu JF, Zhu LZ, Fang XF, Yuan Y (2015). Different treatment strategies and molecular features between right-sided and left-sided colon cancers. World J Gastroenterol.

[24] Tamm I, Wang Y, Sausville E, Scudiero DA, Vigna N, Oltersdorf T, Reed JC (1998). IAP-family protein survivin inhibits caspase activity and apoptosis induced by Fas (CD95), Bax, caspases, and anticancer drugs. Cancer Res.

[25] Altieri DC (2015). Survivin - The inconvenient IAP. Semin Cell Dev Biol.

[26] Rauch A, Hennig D, Schäfer C, Wirth M, Marx C, Heinzel T, Schneider G, Krämer OH (2014). Survivin and YM155: how faithful is the liaison?. Biochim Biophys Acta.

[27] Bertoli C, Skotheim JM, de Bruin RA (2013). Control of cell cycle transcription during G1 and S phases. Nat Rev Mol Cell Biol.

[28] Kurimchak A, Grana X (2015). PP2A: more than a reset switch to activate pRB proteins during the cell cycle and in response to signaling cues. Cell Cycle.

[29] Mimmler M, Peter S, Kraus A, Stroh S, Nikolova T, Seiwert N, Hasselwander S, Neitzel C, Haub J, Monien BH, Nicken P, Steinberg P, Shay JW (2016). DNA damage response curtails detrimental replication stress and chromosomal instability induced by the dietary carcinogen PhIP. Nucleic Acids Res.

[30] Shieh SY, Ahn J, Tamai K, Taya Y, Prives C (2000). The human homologs of checkpoint kinases Chk1 and Cds1 (Chk2) phosphorylate p53 at multiple DNA damage-inducible sites. Genes Dev.

[31] Meek DW (2009). Tumour suppression by p53: a role for the DNA damage response?. Nat Rev Cancer.

[32] Kruiswijk F, Labuschagne CF, Vousden KH (2015). p53 in survival, death and metabolic health: a lifeguard with a licence to kill. Nat Rev Mol Cell Biol.

[33] Podhorecka M, Skladanowski A, Bozko P (2010). H2AX Phosphorylation: Its Role in DNA Damage Response and Cancer Therapy. J Nucleic Acids.

[34] Los M, Mozoluk M, Ferrari D, Stepczynska A, Stroh C, Renz A, Herceg Z, Wang ZQ, Schulze-Osthoff K (2002). Activation and caspase-mediated inhibition of PARP: a molecular switch between fibroblast necrosis and apoptosis in death receptor signaling. Mol Biol Cell.

[35] Toledo LI, Murga M, Zur R, Soria R, Rodriguez A, Martinez S, Oyarzabal J, Pastor J, Bischoff JR, Fernandez-Capetillo O (2011). A cell-based screen identifies ATR inhibitors with synthetic lethal properties for cancer-associated mutations. Nat Struct Mol Biol.

[36] Pons M, Reichardt CM, Hennig D, Nathan A, Kiweler N, Stocking C, Wichmann C, Christmann M, Butter F, Reichardt S, Schneider G, Heinzel T, Englert C (2018 Mar 27). Loss of Wilms tumor 1 protein is a marker for apoptosis in response to replicative stress in leukemic cells. Arch Toxicol.

[37] Schneider G, Henrich A, Greiner G, Wolf V, Lovas A, Wieczorek M, Wagner T, Reichardt S, von Werder A, Schmid RM, Weih F, Heinzel T, Saur D (2010). Cross talk between stimulated NF-kappaB and the tumor suppressor p53. Oncogene.

[38] Stauber RH, Knauer SK, Habtemichael N, Bier C, Unruhe B, Weisheit S, Spange S, Nonnenmacher F, Fetz V, Ginter T, Reichardt S, Liebmann C, Schneider G (2012). A combination of a ribonucleotide reductase inhibitor and histone deacetylase inhibitors downregulates EGFR and triggers BIM-dependent apoptosis in head and neck cancer. Oncotarget.

[39] Schäfer C, Göder A, Beyer M, Kiweler N, Mahendrarajah N, Rauch A, Nikolova T, Stojanovic N, Wieczorek M, Reich TR, Tomicic MT, Linnebacher M, Sonnemann J (2017). Class I histone deacetylases regulate p53/NF-kappaB crosstalk in cancer cells. Cell Signal.

[40] Schneider G, Krämer OH (2011). NFkappaB/p53 crosstalk-a promising new therapeutic target. Biochim Biophys Acta.

[41] Pang LY, Scott M, Hayward RL, Mohammed H, Whitelaw CB, Smith GC, Hupp TR (2011). p21(WAF1) is component of a positive feedback loop that maintains the p53 transcriptional program. Cell Cycle.

[42] Krämer OH, Knauer SK, Zimmermann D, Stauber RH, Heinzel T (2008). Histone deacetylase inhibitors and hydroxyurea modulate the cell cycle and cooperatively induce apoptosis. Oncogene.

[43] Rao S, Beckman RA, Riazi S, Yabar CS, Boca SM, Marshall JL, Pishvaian MJ, Brody JR, Madhavan S (2017). Quantification and expert evaluation of evidence for chemopredictive biomarkers to personalize cancer treatment. Oncotarget.

[44] Zhang LQ, Wang J, Jiang F, Xu L, Liu FY, Yin R (2012). Prognostic value of survivin in patients with non-small cell lung carcinoma: a systematic review with meta-analysis. PLoS One.

[45] Kim K, Chie EK, Wu HG, Kim SG, Lee SH, Kang GH, Hyun CL, Ha SW (2011). High survivin expression as a predictor of poor response to preoperative chemoradiotherapy in locally advanced rectal cancer. Int J Colorectal Dis.

[46] Kawasaki H, Altieri DC, Lu CD, Toyoda M, Tenjo T, Tanigawa N (1998). Inhibition of apoptosis by survivin predicts shorter survival rates in colorectal cancer. Cancer Res.

[47] Blanc-Brude OP, Mesri M, Wall NR, Plescia J, Dohi T, Altieri DC (2003). Therapeutic targeting of the survivin pathway in cancer: initiation of mitochondrial apoptosis and suppression of tumor-associated angiogenesis. Clin Cancer Res.

[48] Carmena M, Wheelock M, Funabiki H, Earnshaw WC (2012). The chromosomal passenger complex (CPC): from easy rider to the godfather of mitosis. Nat Rev Mol Cell Biol.

[49] Koehler BC, Jager D, Schulze-Bergkamen H (2014). Targeting cell death signaling in colorectal cancer: current strategies and future perspectives. World J Gastroenterol.

[50] Hata T, Yamamoto H, Ngan CY, Koi M, Takagi A, Damdinsuren B, Yasui M, Fujie Y, Matsuzaki T, Hemmi H, Xu X, Kitani K, Seki Y (2005). Role of p21waf1/cip1 in effects of oxaliplatin in colorectal cancer cells. Mol Cancer Ther.

[51] Toscano F, Parmentier B, Fajoui ZE, Estornes Y, Chayvialle JA, Saurin JC, Abello J (2007). p53 dependent and independent sensitivity to oxaliplatin of colon cancer cells. Biochem Pharmacol.

[52] Jiang Y, Saavedra HI, Holloway MP, Leone G, Altura RA (2004). Aberrant regulation of survivin by the RB/E2F family of proteins. J Biol Chem.

[53] Wagner T, Kiweler N, Wolff K, Knauer SK, Brandl A, Hemmerich P, Dannenberg JH, Heinzel T, Schneider G, Krämer OH (2015). Sumoylation of HDAC2 promotes NF-κB-dependent gene expression. Oncotarget.

[54] O'Connor DS, Wall NR, Porter AC, Altieri DC (2002). A p34(cdc2) survival checkpoint in cancer. Cancer Cell.

[55] Piret B, Piette J (1996). Topoisomerase poisons activate the transcription factor NF-kappaB in ACH-2 and CEM cells. Nucleic Acids Res.

[56] Esposito D, Crescenzi E, Sagar V, Loreni F, Russo A, Russo G (2014). Human rpL3 plays a crucial role in cell response to nucleolar stress induced by 5-FU and L-OHP. Oncotarget.

[57] Chiu SJ, Lee YJ, Hsu TS, Chen WS (2009). Oxaliplatin-induced gamma-H2AX activation via both p53-dependent and -independent pathways but is not associated with cell cycle arrest in human colorectal cancer cells. Chem Biol Interact.

[58] Konstantinopoulos PA, Ceccaldi R, Shapiro GI, D'Andrea AD (2015). Homologous Recombination Deficiency: Exploiting the Fundamental Vulnerability of Ovarian Cancer. Cancer Discov.

[59] Massey AJ, Stokes S, Browne H, Foloppe N, Fiumana A, Scrace S, Fallowfield M, Bedford S, Webb P, Baker L, Christie M, Drysdale MJ, Wood M (2015). Identification of novel, *in vivo* active Chk1 inhibitors utilizing structure guided drug design. Oncotarget.

[60] Kuehn F, Mullins CS, Krohn M, Harnack C, Ramer R, Kramer OH, Klar E, Huehns M, Linnebacher M (2016). Establishment and characterization of HROC69 - a Crohn s related colonic carcinoma cell line and its matched patient-derived xenograft. Sci Rep.

[61] Maletzki C, Huehns M, Knapp P, Waukosin N, Klar E, Prall F, Linnebacher M (2015). Functional Characterization and Drug Response of Freshly Established Patient-Derived Tumor Models with CpG Island Methylator Phenotype. PLoS One.

[62] Beyer M, Kiweler N, Mahboobi S, Krämer OH (2017). How to Distinguish Between the Activity of HDAC1-3 and HDAC6 with Western Blot. Methods Mol Biol.

[63] Huang X, Darzynkiewicz Z (2006). Cytometric assessment of histone H2AX phosphorylation: a reporter of DNA damage. Methods Mol Biol.

