# Supplementary material for: Survivin antagonizes chemotherapy-induced cell death of colorectal cancer cells
Source: Oncotarget. 2018 Jun 12;9(45):27835–50. doi: 10.18632/oncotarget.25600 (PMC6021236; doi:10.18632/oncotarget.25600)
Supplement: Supplementary file 1 [file oncotarget-09-27835-s001.pdf]

Survivin antagonizes chemotherapy-induced cell death of colorectal cancer cells

SUPPLEMENTARY MATERIALS

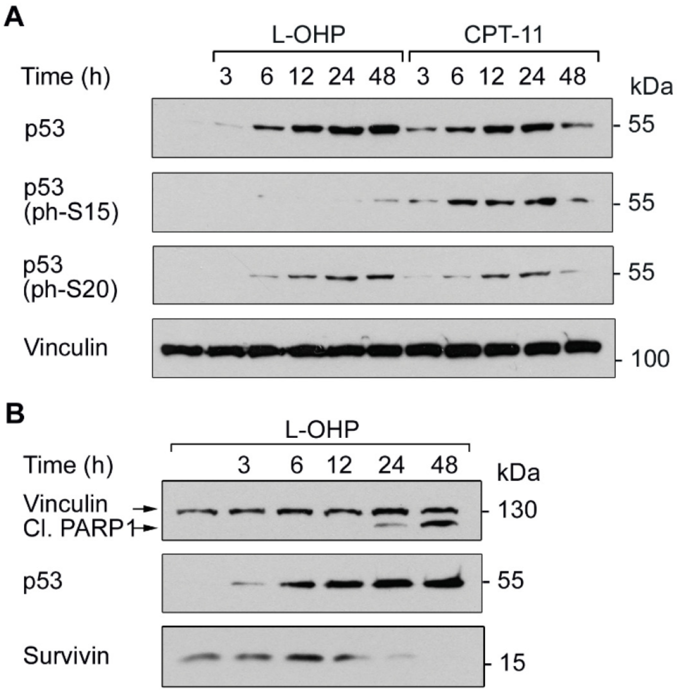

Supplementary Figure 1: Time-dependent effects of L-OHP and CPT-11. HCT116 cells were treated for the indicated times with 5 µM CPT-11 or 10 µM CPT-11 (n=3). **(A)** Western blot analysis shows p53 phosphorylation at S15 and S20. **(B)** Immunodetection of cleaved PARP1 (cl.), p53, and survivin; vinculin serves as loading control.

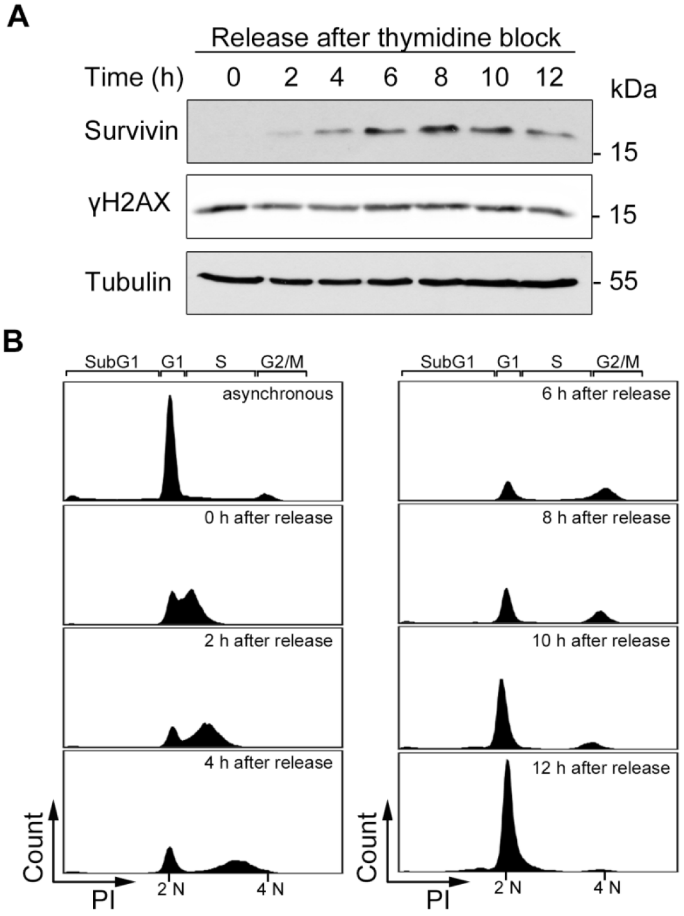

Supplementary Figure 2: Regulation of survivin during cell cycle progression. HCT116 cells were arrested by double-thymidine block, followed by a release in fresh culture medium for up to 12 hours. **(A)** Immunodetection of survivin and γH2AX protein levels; α-tubulin serves as loading control. **(B)** Cell cycle distributions as determined by PI staining and flow cytometric analysis of DNA contents (n = 3).

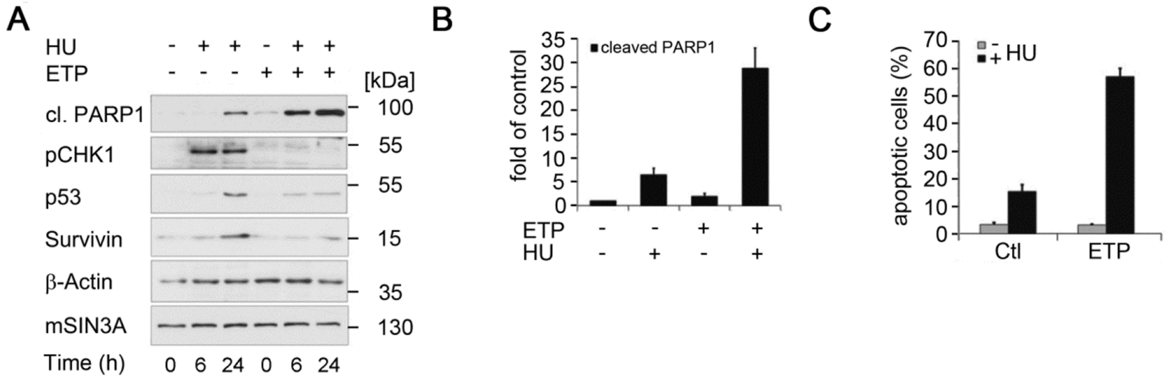

Supplementary Figure 3: Impact of ATR activity on hydroxyurea-induced survivin levels. (A) HCT116 cells were pre-treated with 3 µM ETP-46464 for 1 hour following treatment with 1 mM hydroxyurea for 6 to 24 hours (Ctl, untreated). Expression of cleaved PARP, p53, pCHK1, and Survivin was determined by Western blot; β-actin and mSIN3A are loading controls. **(B)** Quantification of cleaved PARP signal detected by western blot. The graph displays average results from two independent experiments. **(C)** HCT116 cells were pre-treated with 3 µM ETP-46464 for 1 hour following incubation with 1 mM hydroxyurea for 48 hours. Apoptosis rate was determined by annexin-V staining and flow cytometry. The graph shows average results from three independent experiments.

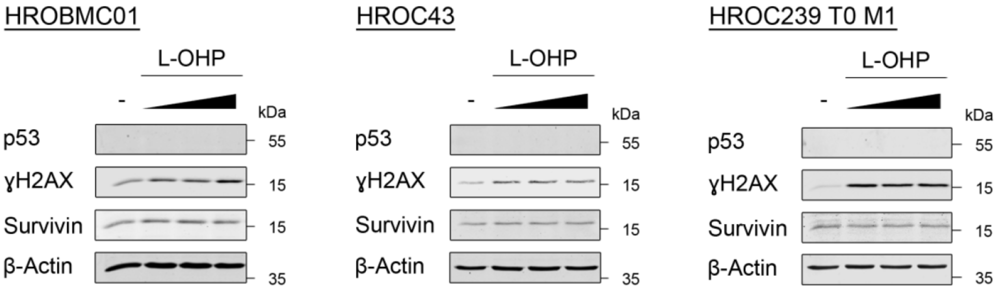

Supplementary Figure 4: Lack of p53 associated with persistence of survivin in L-OHP-treated cells. Three p53-negative colon cancer cells (HROBMC01, HROC43, HROC239) were treated with DMSO (-), 2.5, 5 or 10 µM L-OHP for 24 h. Western blot analysis revealed p53, γH2AX, survivin, and β-actin (loading control).
